# Supplementary material for: Brain meta-state transitions demarcate thoughts across task contexts exposing the mental noise of trait neuroticism
Source: Nat Commun. 2020 Jul 13;11:3480. doi: 10.1038/s41467-020-17255-9 (PMC7359033; doi:10.1038/s41467-020-17255-9)
Supplement: Supplementary file 1 — Supplementary Information [file 41467_2020_17255_MOESM1_ESM.pdf]

Brain meta-state transitions demarcate thoughts across task contexts exposing the mental noise of trait neuroticism

Tseng & Poppenk

Table of Contents

Supplementary Figure 1. Expression of brain network activity……………………………………2

Supplementary Figure 2. Comparison of noise and real mean step distance vectors……………3

Supplementary Figure 3. Effects of varying peak threshold and smoothing spans……………….4

Supplementary Figure 4. Impact of embedding approach on transitions' movie alignment……...5

Supplementary Figure 5. Higher transition rate correlated to higher trait neuroticism……………6

Supplementary Figure 6. Correlations between 3T transition rate and personality………………7

Supplementary Table 1. Voxel clusters in which activation reliability distinguished transition from meta-stable timepoints specifically during movie-viewing fMRI……………………………………..8

Supplementary Table 2. Voxel clusters in which activation reliability distinguished transition from meta-stable timepoints specifically during resting state fMRI………………………………………..9

Supplementary Table 3. Voxel clusters in which activation reliability distinguished transition from meta-stable timepoints in both movie-viewing and resting state fMRI……………………………10

Supplementary Figures

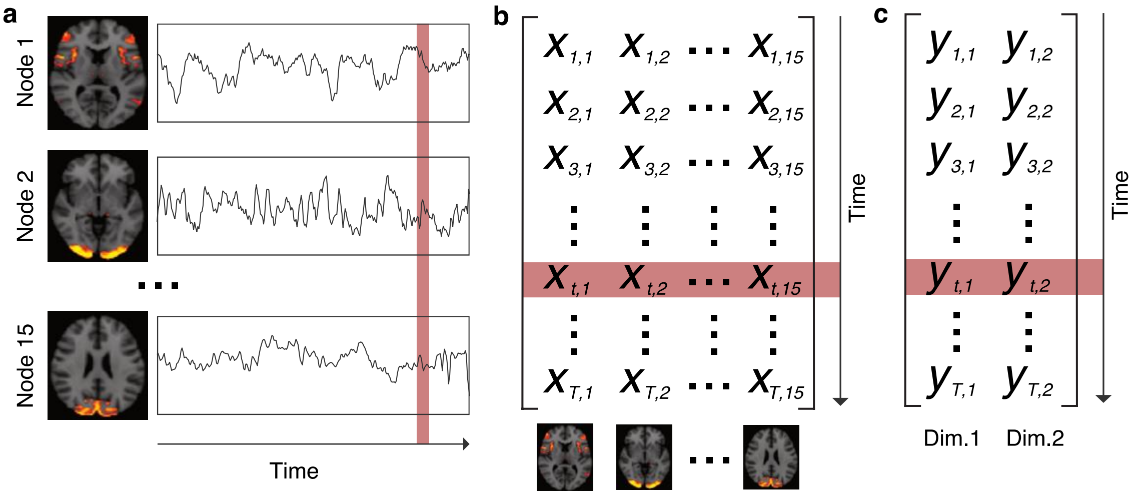

Supplementary Figure 1. Expression of brain network activity. (**a**) Each fMRI run of each participant was characterized as the activation of 15 brain networks over time. (**b**) Representation of A as a two-dimensional matrix of (time x network). (**c**) Reduced representation obtained after applying t-SNE algorithm. (**a-c**) Red bar defining a specific TR across all three representations.

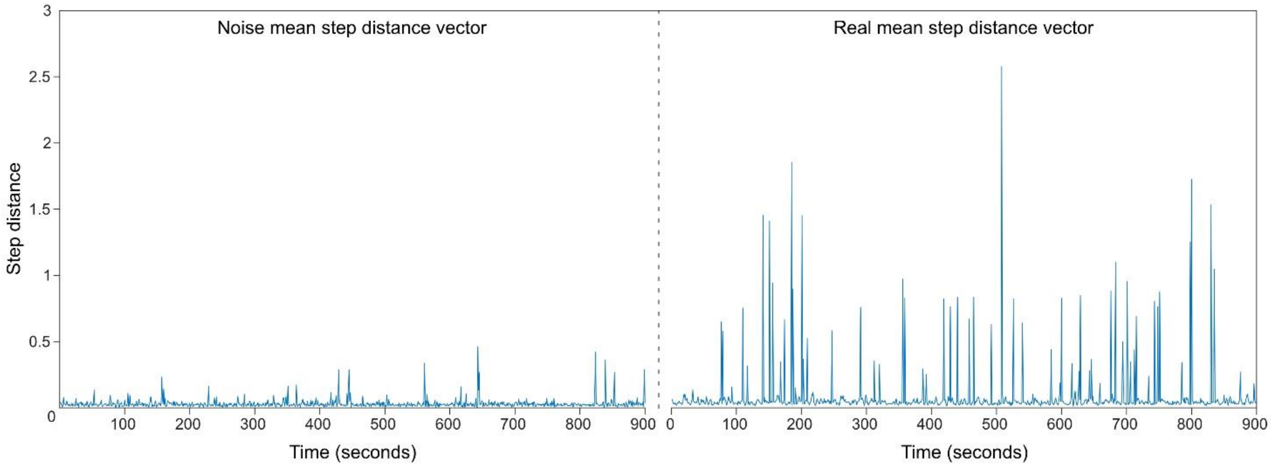

Supplementary Figure 2. Comparison of noise and real mean step distance vectors. A noise dataset was generated by phase randomizing in a voxel-wise fashion each participant's Movie 1 fMRI run. The same procedure (dual regression, 100 iterations of the t-SNE algorithm, Mahalanobis distance calculation) was carried out to obtain mean noise step distance vectors. One participant's noise vs. real step distance vector is shown above, revealing that t-SNE space representations of noise data do not jump at consistent timepoints across repeated iterations, resulting in smaller transition amplitudes than in the real step distance vector.

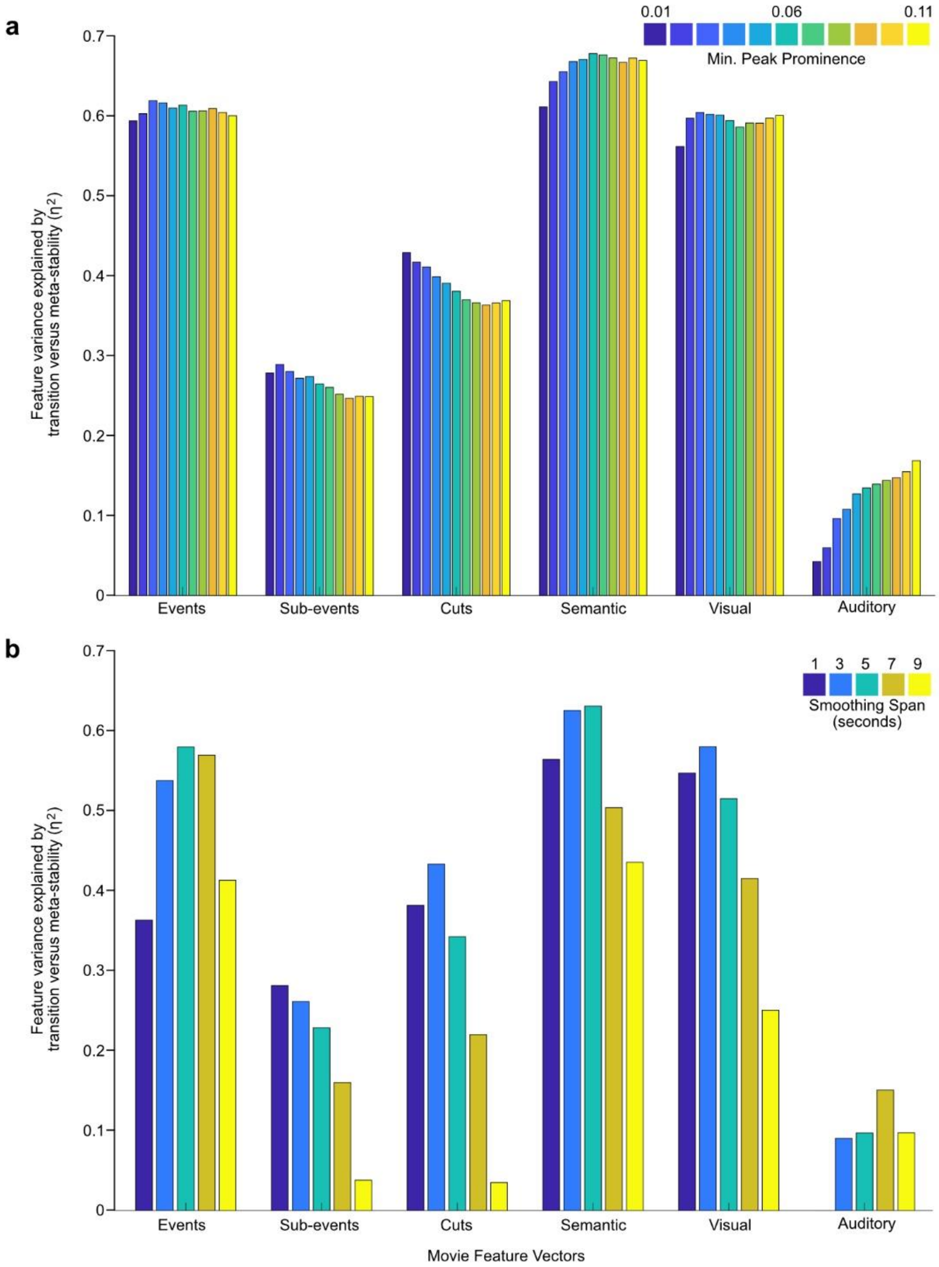

Supplementary Figure 3. Effects of varying peak threshold and smoothing spans. Eta-squared values describing the proportion of variance in movie features explained by alignment to transition vs. meta-stable timepoints. The colour scale from blue to yellow denotes increasing parameter values. Note that all auditory feature results showed stronger association to meta-stable rather than transition timepoints. (**a**) Comparison of eta-squared results for varying minimum peak prominence. (**b**) Comparison of eta-squared results for varying temporal smoothing spans.

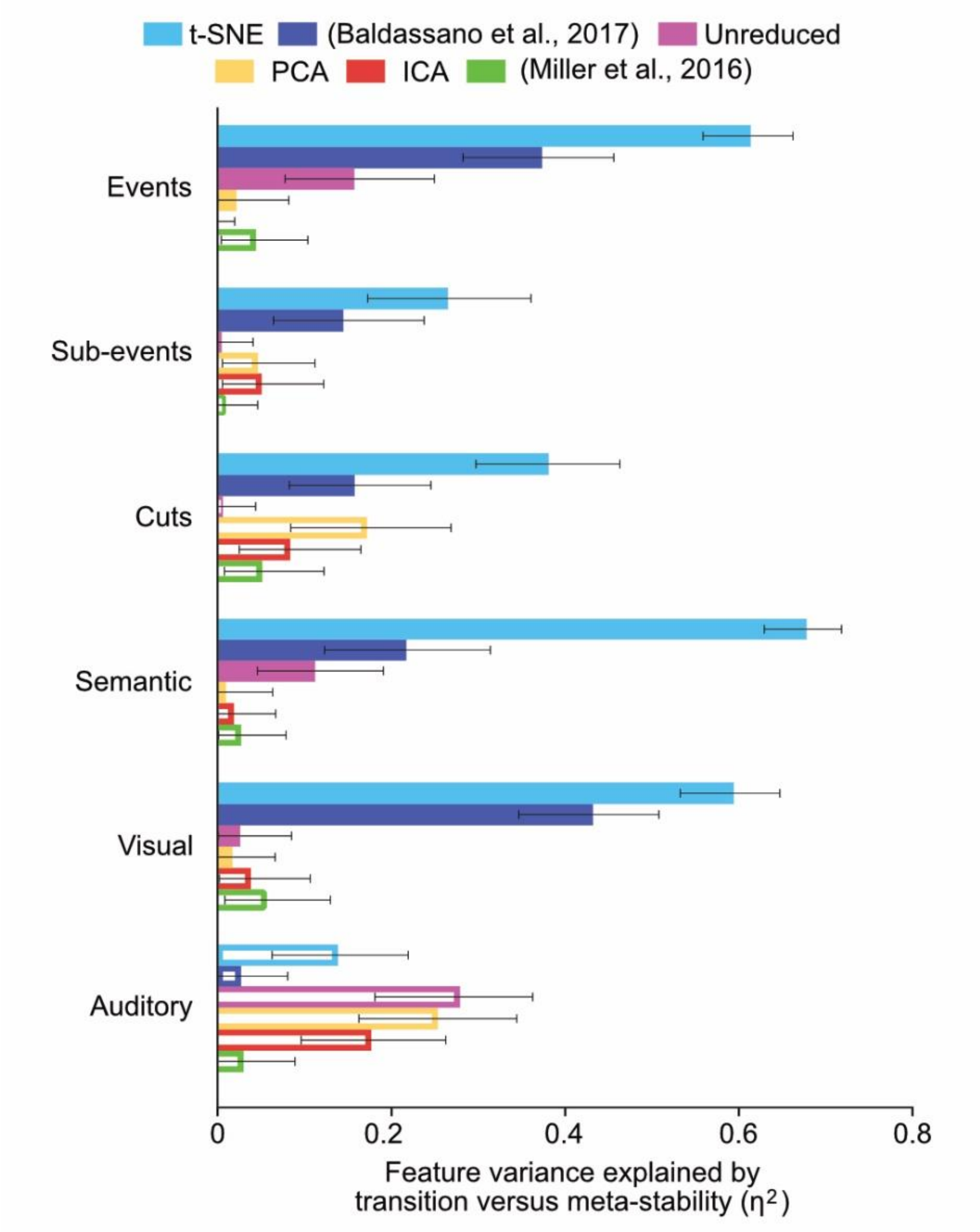

Supplementary Figure 4. Impact of embedding approach on transitions' movie feature alignment. Data are presented as eta-squared values describing the proportion of variance in movie features explained by alignment to transition vs. meta-stable timepoints. See Figure 3 for a description of the calculation procedure (n = 184). Colours indicate the method used to identify transition and meta-stable timepoints. Filled bars denote features that are aligned to transitions, whereas empty bars denote stronger alignment to meta-stability. Error bars designate eta-squared values corresponding to 95% percentile bootstrap confidence intervals of the transition-meta-stable feature value difference.

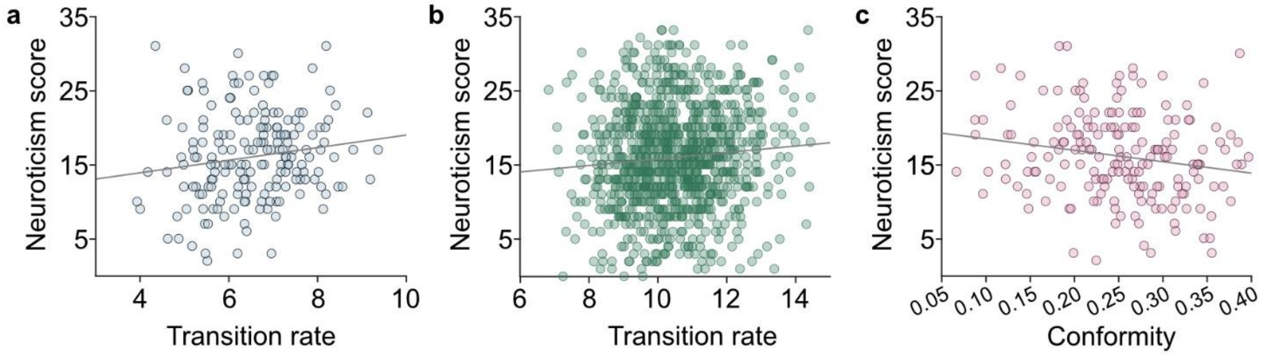

Supplementary Figure 5. Higher transition rate correlated to higher trait neuroticism. Transition rate is calculated as the total number of transitions divided by the time in minutes. Neuroticism scores are obtained from the NEO Five Factor Inventory administered to HCP participants. (**a**) Correlation between transition rate and neuroticism in the 7T dataset. (**b**) Correlation between transition rate and neuroticism in the larger 3T dataset. (**c**) Correlation between conformity and neuroticism in the 7T dataset.

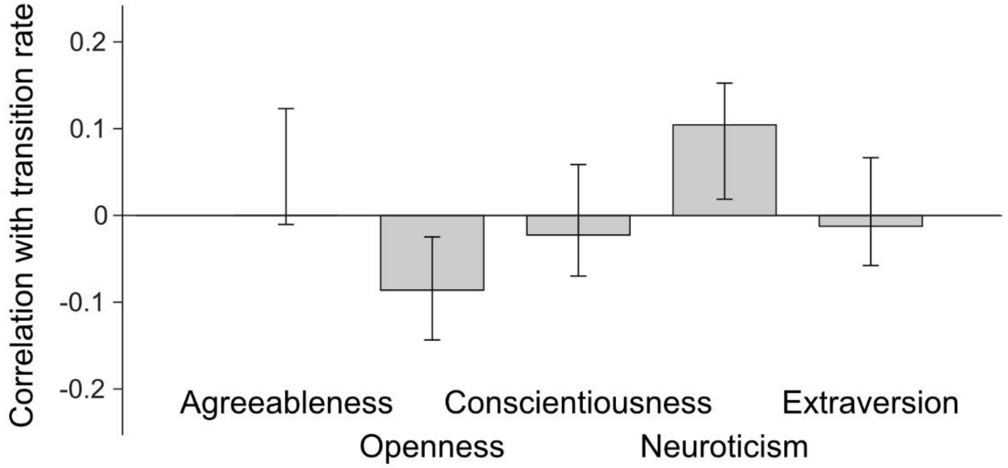

Supplementary Figure 6. Correlations between 3T transition rate and personality. Exploratory results here are presented as correlation values between resting state transition rate and personality traits, obtained through bootstrapping procedures with 1,000 samples (n = 1003). Error bars designate 95% percentile bootstrap confidence intervals.

Supplementary Table 1. Voxel clusters in which activation reliability distinguished transition from meta-stable timepoints specifically during movie-viewing fMRI.

Region	BA	Hemi.	Peak MNI coordinates			Peak *z* estimate	Spatial extent (mm³)
			X	Y	Z		
**Movie: Transition > Meta-stable**							
Frontal lobe							
Ventromedial prefrontal ctx.	11	L/R	-11	64	-19	4.50	3531
	11	L/R	0	22	-29	3.99	1802
Dorsal anterior cingulate	32	L/R	-2	26	27	6.53	3998
Insular lobe							
Insula	13	L	-35	11	11	5.21	1331
Parietal lobe							
Posterior cingulate	23	L/R	-2	-38	22	7.84	2679
Dorsal posterior cingulate	31	L/R	11	-38	51	7.72	3596
Temporal lobe							
Middle temporal g.	20/21	R	48	-16	-29	4.35	2281
Visual lobe							
Visual assoc. area.	17/18	L/R	0	-80	32	7.87	13431
Cerebellum							
Cerebellum	-	L/R	-2	-78	-34	4.83	11788
**Movie: Meta-stable > Transition**							
Frontal lobe							
Lateral prefrontal ctx.	46	L	-46	45	2	-5.48	2044
	47	R	45	42	-5	-6.41	4461
Dorsomedial prefrontal ctx.	8/9/10	L/R	-2	32	46	-7.68	4784
	6/44	L	-48	22	24	-5.97	4923
	8	R	42	22	40	-5.18	1200
	6/8	L	26	16	51	-5.40	1241
Dorsolateral prefrontal ctx.	6/44/46	R	51	10	19	-5.95	6222
Temporal lobe							
Temporal pole / posterior ventromedial prefrontal ctx. / amygdala / thalamus	38	L/R	35	16	-21	-5.67	15462
Parietal lobe							
Precuneus	7	L	-43	-43	48	-4.95	1368
Supramarginal g.	40	R	48	-34	50	-5.26	2621
Angular g.	39	R	54	-53	38	-4.87	3011
	39	L	-54	-58	29	-5.82	4055
Visual lobe							
Visual assoc. area	18	R	11	-91	-2	-6.01	6328

Supplementary Table 2. Voxel clusters in which activation reliability distinguished transition from meta-stable timepoints specifically during resting state fMRI.

Region	BA	Hemi.	Peak MNI coordinates			Peak *z* estimate	Spatial extent (mm³)
			X	Y	Z		
**Rest: Transition > Meta-stable**							
Frontal / parietal lobe							
Cingulate ctx.	7/23/32	L/R	2	-29	27	11.02	33243
Somatosensory ctx.	1	R	30	-30	62	5.06	1671
Supramarginal g.	40	R	54	-32	29	7.58	6644
Insular lobe							
Insula	13	R	46	13	-3	7.24	1548
Visual lobe							
Visual assoc ctx.	7/17/18	L/R	-10	-77	37	13.03	39973
Subcortical							
Cerebellum	-	L	-32	-51	-26	4.58	1200
**Rest: Meta-stable > Transition**							
Frontal lobe							
Lateral prefrontal ctx.	47	L	-43	45	-5	-5.08	1110
	44	L	-51	21	6	-6.86	2765
Dorsolateral prefrontal ctx.	6	L	-24	18	53	-6.24	11010
	6	R	32	24	58	-6.13	2585
Temporal lobe							
Middle temporal g.	20	R	54	0	-34	-4.64	1253
Parietal lobe							
Supramarginal g.	40	L	-46	-43	48	-6.29	6250

Supplementary Table 3. Voxel clusters in which activation reliability distinguished transition from meta-stable timepoints in both movie-viewing and resting state fMRI.

Region	BA	Hemi.	Peak MNI coordinates			Peak *z* estimate	Peak z (rest)	Peak z (movie)	Spatial extent (mm³)
			X	Y	Z				
**Transition > Meta-stable**									
Frontal lobe									
Dorsal anterior cingulate ctx.	32	L/R	-2	18	38	9.11	7.81	4.35	8327
Insular lobe									
Insula	13	L	-35	10	11	6.76	4.08	5.11	2208
	13	R	45	13	-3	9.28	7.22	5.52	2556
Parietal lobe									
Supramarginal g.	40	R	54	-30	32	7.81	7.20	2.59	1028
Posterior cingulate ctx.	23	L/R	0	-37	24	11.17	7.99	7.55	3858
Precuneus	7	L/R	11	-38	51	9.38	5.00	7.72	10572
Visual lobe									
Visual assoc. ctx.	7/18/19	L/R	0	-80	32	11.54	8.19	7.87	24965
Subcortical									
Cerebellum	-	L/R	-5	-74	-13	6.10	3.44	4.77	1995
**Meta-stable > Transition**									
Frontal lobe									
Lateral prefrontal ctx.	47	L	-43	45	-3	-6.33	-4.90	-3.65	1712
	44	L	-45	11	32	-7.46	-4.96	-5.29	5272
	8	R	40	24	38	-6.47	-4.12	-4.71	1651
Dorsomedial prefrontal ctx.	8	L/R	-2	32	45	-7.84	-2.60	-7.23	1716
Dorsal prefrontal ctx.	6/8	L	-24	18	51	-6.68	-5.95	-2.61	1499
	6/8	R	26	16	51	-7.42	-4.79	-5.40	2830
Parietal lobe									
Superior parietal l.	7	L	-45	-43	48	-7.93	-6.09	-4.76	8897
Angular g.	39	R	46	-59	32	-6.39	-5.62	-2.61	1098
