# Supplementary material for: Brain meta-state transitions demarcate thoughts across task contexts exposing the mental noise of trait neuroticism
Source: Nat Commun. 2020 Jul 13;11:3480. doi: 10.1038/s41467-020-17255-9 (PMC7359033; doi:10.1038/s41467-020-17255-9)
Supplement: Supplementary file 3 — Reporting Summary [file 41467_2020_17255_MOESM3_ESM.pdf]

nature research

Corresponding author(s): Julie Tseng
Jordan Poppenk

Last updated by author(s): April 9, 2020

# Reporting Summary

Nature Research wishes to improve the reproducibility of the work that we publish. This form provides structure for consistency and transparency in reporting. For further information on Nature Research policies, see Authors & Referees and the Editorial Policy Checklist.

## Statistics

For all statistical analyses, confirm that the following items are present in the figure legend, table legend, main text, or Methods section.

| n/a | Confirmed | |
|---|---|---|
| ☐ | ☒ | The exact sample size (*n*) for each experimental group/condition, given as a discrete number and unit of measurement |
| ☐ | ☒ | A statement on whether measurements were taken from distinct samples or whether the same sample was measured repeatedly |
| ☐ | ☒ | The statistical test(s) used AND whether they are one- or two-sided<br>*Only common tests should be described solely by name; describe more complex techniques in the Methods section.* |
| ☐ | ☒ | A description of all covariates tested |
| ☐ | ☒ | A description of any assumptions or corrections, such as tests of normality and adjustment for multiple comparisons |
| ☐ | ☒ | A full description of the statistical parameters including central tendency (e.g. means) or other basic estimates (e.g. regression coefficient) AND variation (e.g. standard deviation) or associated estimates of uncertainty (e.g. confidence intervals) |
| ☐ | ☒ | For null hypothesis testing, the test statistic (e.g. $F$, $t$, $r$) with confidence intervals, effect sizes, degrees of freedom and $P$ value noted<br>*Give P values as exact values whenever suitable.* |
| ☒ | ☐ | For Bayesian analysis, information on the choice of priors and Markov chain Monte Carlo settings |
| ☒ | ☐ | For hierarchical and complex designs, identification of the appropriate level for tests and full reporting of outcomes |
| ☐ | ☒ | Estimates of effect sizes (e.g. Cohen's $d$, Pearson's $r$), indicating how they were calculated |

*Our web collection on statistics for biologists contains articles on many of the points above.*

## Software and code

Policy information about availability of computer code

| | |
|---|---|
| Data collection | As we performed an archival analysis, we did not use software for data collection. |
| Data analysis | We performed analysis using MATLAB R2017a, FreeSurfer v5.3, Datavyu v1.3.7, and FSL v5.0.10, BrainIAK (RRID: SCR_014824), R v3.6.2, and have made our analysis code available on Github at https://github.com/j-tseng/neural-transitions |

For manuscripts utilizing custom algorithms or software that are central to the research but not yet described in published literature, software must be made available to editors/reviewers. We strongly encourage code deposition in a community repository (e.g. GitHub). See the Nature Research guidelines for submitting code & software for further information.

## Data

Policy information about availability of data

All manuscripts must include a data availability statement. This statement should provide the following information, where applicable:

- Accession codes, unique identifiers, or web links for publicly available datasets
- A list of figures that have associated raw data
- A description of any restrictions on data availability

Data are available from the Human Connectome Project at humanconnectome.org. Movie-related variables (e.g., event boundaries segmented for this study) are available upon request.

# Field-specific reporting

Please select the one below that is the best fit for your research. If you are not sure, read the appropriate sections before making your selection.

☐ Life sciences ☒ Behavioural & social sciences ☐ Ecological, evolutionary & environmental sciences

For a reference copy of the document with all sections, see nature.com/documents/nr-reporting-summary-flat.pdf

# Behavioural & social sciences study design

All studies must disclose on these points even when the disclosure is negative.

| | |
|---|---|
| Study description | This study applies new methods that detects boundaries between thoughts in a healthy, young adult without priors. The study attempts to validate the psychological properties of this method by relating transition onsets to movie features, and transition rate to neuroticism. It also investigates the neural correlates of transitions and their generalizability across task contexts. |
| Research sample | This study analyzed the Human Connectome Project Young Adult 7T and 3T datasets. For the 7T dataset, there were 184 participants between the ages of 22 and 36 (age M = 29.4, SD = 3.4 years, 112 female) with both movie-viewing and resting fMRI scans. A complete set of data would consist of four 15-minute resting state fMRI runs, four approx. 15-minute movie-viewing functional runs scanned with a 7T MRI. For the 3T dataset, there were 1003 participants with the same age range (age M = 28.7 years, SD = 3.7 years; 534 female) with only resting fMRI runs. A complete set of data would consist of four approx. 15-minute resting state functional runs scanned with a 3T MRI (no movie-viewing). All participants in both datasets had neuroticism results from the 60-item version of the Costa and McRae Neuroticism/Extroversion/Openness Five Factor Inventory (NEO-FFI). We selected the 7T dataset for its large N and availability of both movie and resting fMRI data for the same participants, allowing ready comparison. We selected the 3T dataset for its especially large N, even though it could not be used in all analyses due to the absence of movie data. |
| Sampling strategy | The entire archival sample was utilized. |
| Data collection | A 7T and 3T fMRI scanner was used to gather brain data. Data were gathered by another group that was unaware of our intended use. Details of data acquisition are available at humanconnectome.org. |
| Timing | August 2012 to October 2015 |
| Data exclusions | No participants were excluded. |
| Non-participation | The 7T HCP study aimed to scan 200 participants (Ugurbil et al., 2013), but distributed data from only 184. Reasons for this discrepancy were not reported. We did not ourselves exclude any participants. |
| Randomization | Our analysis did not involve any groups. We controlled for intracranial volume (ICV) in our individual differences analysis. |

# Reporting for specific materials, systems and methods

We require information from authors about some types of materials, experimental systems and methods used in many studies. Here, indicate whether each material, system or method listed is relevant to your study. If you are not sure if a list item applies to your research, read the appropriate section before selecting a response.

## Materials & experimental systems

| n/a | Involved in the study | |
|---|---|---|
| ☒ | ☐ | Antibodies |
| ☒ | ☐ | Eukaryotic cell lines |
| ☒ | ☐ | Palaeontology |
| ☒ | ☐ | Animals and other organisms |
| ☐ | ☒ | Human research participants |
| ☒ | ☐ | Clinical data |

## Methods

| n/a | Involved in the study | |
|---|---|---|
| ☒ | ☐ | ChIP-seq |
| ☒ | ☐ | Flow cytometry |
| ☐ | ☒ | MRI-based neuroimaging |

## Human research participants

Policy information about studies involving human research participants

| | |
|---|---|
| Population characteristics | 7T dataset: 184 participants (age M = 29.4 years, SD = 3.4 years; 112 female. 3T dataset: 1003 participants (age M = 28.7 years, SD = 3.7 years; 534 female). |
| Recruitment | See overview of WU-Minn Human Connectome Project (Van Essen et al., 2013). |

Ethics oversight | Our use of the archival dataset was approved by the Health Sciences Research Ethics Board at Queen's University.

Note that full information on the approval of the study protocol must also be provided in the manuscript.

# Magnetic resonance imaging

## Experimental design

Design type | Resting state, movie-viewing

Design specifications | Four resting-state runs and four movie-viewing runs were distributed equally across four MRI sessions, with each run lasting approximately 15 minutes.

Behavioral performance measures | Although the HCP consortium gathered a variety of behavioral measures, these responses were not relevant to our analysis. Rather, our analyses were responsive to the degree tasks elicited participant engagement.

## Acquisition

Imaging type(s) | Functional

Field strength | 7 Tesla

Sequence & imaging parameters | Multiband gradient echo-planar imaging (EPI) pulse sequence, TR 1000 ms, TE 22.2 ms, flip angle 45, multiband factor 5, whole-brain coverage 85 slices of 1.6 mm thickness, in-plane resolution 1.6 x 1.6 mm^2, FOV 208 x 208 mm^2

Area of acquisition | Whole brain scan

Diffusion MRI ☐ Used ☒ Not used

## Preprocessing

Preprocessing software | The HCP "Minimal Preprocessing Scripts" were applied to the data by the HCP group (Glasser et al., 2013) prior to data distribution. These relied on algorithms implemented by FSL (v5.0.6) and Freesurfer (v5.3.0-HCP).

Normalization | Linear trend removal and intensity normalization were applied, and images were volumetrically registered to standard MNI space, then surface registration to Conte69 mesh.

Normalization template | MNI152 (non-linear registration to standard MNI space for each participant), Conte69 '164k_fs_LR' mesh (surface registration).

Noise and artifact removal | ICA-FIX was applied for purposes of noise / artifact removal.

Volume censoring | No volume censoring was performed.

## Statistical modeling & inference

Model type and settings | Multiple used; our full-brain analysis employed a mass univariate model using a mixed-effects approach.

Effect(s) tested | A conjunction analysis was performed, evaluating the meta-state transitions > meta-stability effect separately for movie and rest data, as well as evaluating voxels that were stable in both contrasts.

Specify type of analysis: ☐ Whole brain ☐ ROI-based ☒ Both

Anatomical location(s) | Anterior and posterior hippocampus (Freesurfer-based segmentations were segmented into anterior and posterior parts by expert raters).

Statistic type for inference (See Eklund et al. 2016) | For the conjunction analysis, we used cluster-wise inference with an implied voxel-wise threshold of P=0.0025 and minimum extent of 614mm^3, parameters that have previously been advocated as striking a balance between Type I and Type II error rate.

Correction | Consistent with the position advocated by Lieberman and Cunningham (2009), we opted to apply the above cluster parameters rather than a correction approach.

## Models & analysis

| n/a | Involved in the study |
|---|---|
| ☐ | ☒ Functional and/or effective connectivity |
| ☒ | ☐ Graph analysis |
| ☐ | ☒ Multivariate modeling or predictive analysis |

| | |
|---|---|
| Functional and/or effective connectivity | We employed FSL's dual regression function, a method in which known spatial configurations are regressed against new data to transform 4D functional data into a set of timeseries. |
| Multivariate modeling and predictive analysis | Network templates were obtained from a prior analysis (Nickerson et al., 2017) which involved using group-ICA and group-PCA on the larger 3T dataset. |
